# Synthesis of bi- and bis-1,2,3-triazoles by copper-catalyzed Huisgen cycloaddition: A family of valuable products by click chemistry

**DOI:** 10.3762/bjoc.11.276

**Published:** 2015-12-11

**Authors:** Zhan-Jiang Zheng, Ding Wang, Zheng Xu, Li-Wen Xu

**Affiliations:** 1 Key Laboratory of Organosilicon Chemistry and Material Technology of Ministry of Education, and College of Material, Chemistry and Chemical Engineering, Hangzhou Normal University, Hangzhou 310012, P. R. China; 2 State Key Laboratory for Oxo Synthesis and Selective Oxidation, Lanzhou Institute of Chemical Physics, Chinese Academy of Sciences, Lanzhou, P. R. China

**Keywords:** bistriazoles, click chemistry, cycloaddition, homogeneous catalysis, oxidative coupling

## Abstract

The Cu(I)-catalyzed azide-alkyne cycloaddition reaction, also known as click chemistry, has become a useful tool for the facile formation of 1,2,3-triazoles. Specifically, the utility of this reaction has been demonstrated by the synthesis of structurally diverse bi- and bis-1,2,3-triazoles. The present review focuses on the synthesis of such bi- and bistriazoles and the importance of using copper-promoted click chemistry (CuAAC) for such transformations. In addition, the application of bitriazoles and the related CuAAAC reaction in different fields, including medicinal chemistry, coordination chemistry, biochemistry, and supramolecular chemistry, have been highlighted.

## Introduction

**Scheme 1:**
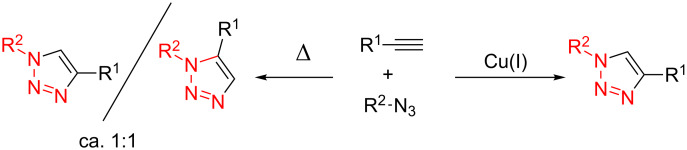
The synthesis of triazoles through the Huisgen cycloaddition of azides to alkynes.

Since its discovery by Huigsen and co-workers fifty years ago [1–4], the Huisgen cycloaddition of azides to alkynes has gained much attention due to its potential to yield a wide variety of triazoles with structurally diverse and functionalized groups, especially with respect to biological activity [5–8]. Originally, this transformation was typically carried out at high temperature and resulted in a mixture of the 1,4 and 1,5 regioisomers (Scheme 1). Fortunately, representing a milestone in this field, the application of Cu(І) as the catalyst was reported by Sharpless and Meldal in 2002 [9–10]. In this work, the Huisgen reaction worked well under mild conditions, giving the desired triazoles with high yield, good regioselectivity, and quite high functional-group tolerances (Scheme 1). Since then, the so-called field of “click chemistry” has been extensively investigated and recognized as an epoch-making progress in organic synthesis and green chemistry [11–15].

After many years of research, it was proven that the Cu(I)-catalyzed azide-alkyne cycloaddition (CuAAC reaction) could be performed under various conditions according to the need of click chemistry using the catalysis of various copper salts that generate Cu(I) sources in situ [16]. As a brief summary, the copper(I)-promoted click chemistry has the following features: (1) The most preferred methods for the formation of Cu(I) involve the use of CuSO_4_ and a reducing agent in an aqueous solution. Most commonly, a large excess of sodium ascorbate with respect to the copper catalyst is the favored reducing agent, and a water/alcohol mixture is the favored solvent. This catalyst system combines the advantage of not requiring inert gas to prevent the Cu(I) from oxidation to Cu(II) during the reaction, and not requiring the tedious work-up and purification steps. (2) Other frequently used Cu(I) sources are CuI or CuBr. In general, CuI has been used in organic solvents such as CH_3_CN, THF or toluene, and the active Cu(I) species was further stabilized by the addition of excess base. In addition, many other Cu(I) salts are used in CuAAC reactions owing to improved solubility or increased rate as compared to the CuSO_4_/sodium ascorbate or CuI catalytic system. (3) The third type of Cu(I) source is generated by the oxidation of Cu metal. The Cu(0) species (found in forms such as turnings, wire, powder or nanoparticles) in the presence or absence of Cu(II) in aqueous media also provides the key active Cu(I) in some CuAAC reactions.

As an important supplement to the classic Huisgen cycloaddition promoted by copper catalysis, this review will deal with the copper-catalyzed syntheses of bi- and bistriazoles or their analogues by click chemistry, such as those linked directly or by spacers. The synthetic approaches for the preparation of bi- and bistriazoles are discussed in detail and their application is discussed briefly in each section. Accordingly, the following three types of bi- and bistriazoles will be primarily presented: (1) The 4,4'-linked symmetric or unsymmetrical bitriazoles. (2) The 5,5'-linked symmetric bitriazoles. (3) The bistriazoles formed through spacers from the dialkyne or diazide substrates.

## Review

### The synthesis of 4,4'-bitriazoles

The 4,4'-bitriazoles can be realized from a double CuAAC reaction between various sources of 1,3-butadiynes with the substituted azides. In general, two different methods have been developed for the construction of the 4,4'-bitriazoles: (1) The one-pot double CuAAC reaction of 1,3-butadiyne with azides. (2) Two successive CuAAC reactions with different or same azides that require the deprotection of the second reactive site to liberate another alkyne moiety.

**Scheme 2:**
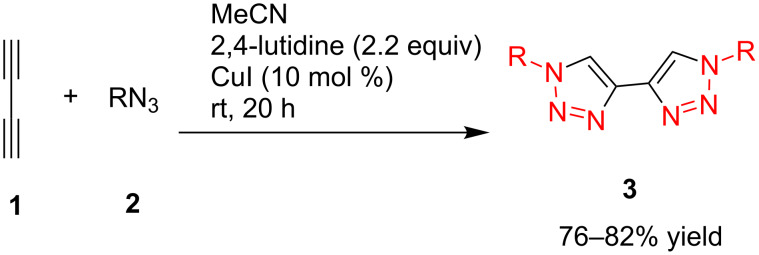
The synthesis of symmetrically substituted 4,4'-bitriazoles.

In 2007, Monkowius et al. reported that the 4,4'-bitriazoles **3** could be synthesized by the two-fold click reaction between 1,3-butadiyne and substituted organic azides [17], and the reaction was catalyzed by a catalytic amount of CuI in acetonitrile in the presence of 2,4-lutidine. The desired, symmetrical 4,4^’^-bitriazoles **3** were obtained in good yield (76–82%, Scheme 2). However, the substrate 1,3-butadiyne (**1**) is difficult to handle because of its high reactivity and instability.

**Scheme 3:**
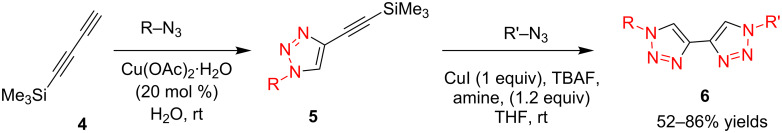
The synthesis of unsymmetrically substituted 4,4'-bitriazoles.

Later in 2009, Fiandanese et al. proposed that unsymmetrically substituted 4,4'-bi-1,2,3-triazoles can be prepared in an easy manner [18]: starting from 1-trimethylsilyl-1,3-butadiyne (**4**) the 4-(silylalkynyl)-1,2,3-triazoles **5** can be obtained after reaction with substituted azides catalyzed by Cu(OAc)_2_·H_2_O (0.2 equiv). After the in situ deprotection with TBAF, followed by the CuI-catalyzed click reaction with another azide in THF in the presence of 1.2 equiv of 1,1,4,7,7-pentamethyldiethylenetriamine, the unsymmetrically 4,4'-bitriazoles **6** are obtained in good yield (52–86%, Scheme 3).

**Scheme 4:**
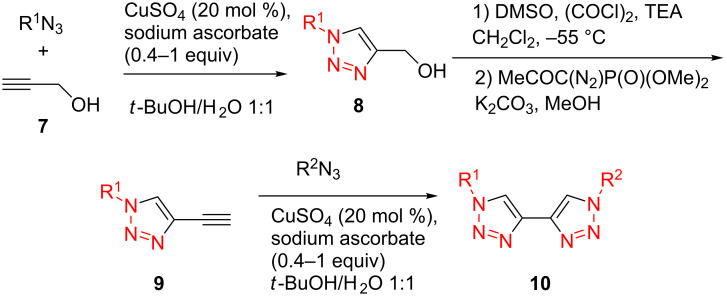
The stepwise preparation of unsymmetrical 4,4'-bitriazoles.

Simpson et al. also developed this three-step procedure (CuAAC–deprotection–CuAAC) into a one-pot fashion with moderate overall yield (34–49%) [19]. Similar to Fiandanese’s strategy, Aizpurua et al. developed another synthetic method [20]: Starting with the CuAAC reaction of propargyl alcohol (**7**) with different azides, followed by the sequential Swern oxidation and Ohira–Bestmann homologation provided the ethynyltriazole intermediate **9**, finally another CuAAC resulted in the formation of unsymmetrical 4,4'-bi(1,2,3-triazole)s **10** (Scheme 4).

### The synthesis of 5,5'-bitriazoles

Originally, in the research of the CuAAC reaction, the 5,5'-bitriazoles were usually considered as an undesired side product or impurity in the Huisgen cycloaddition. In general, they are the oxidative coupling product of the triazole-copper species. The 5,5'-bitriazoles were usually formed as the major product by the facilitation of the reaction conditions or controlled by the starting substrate.

**Scheme 5:**
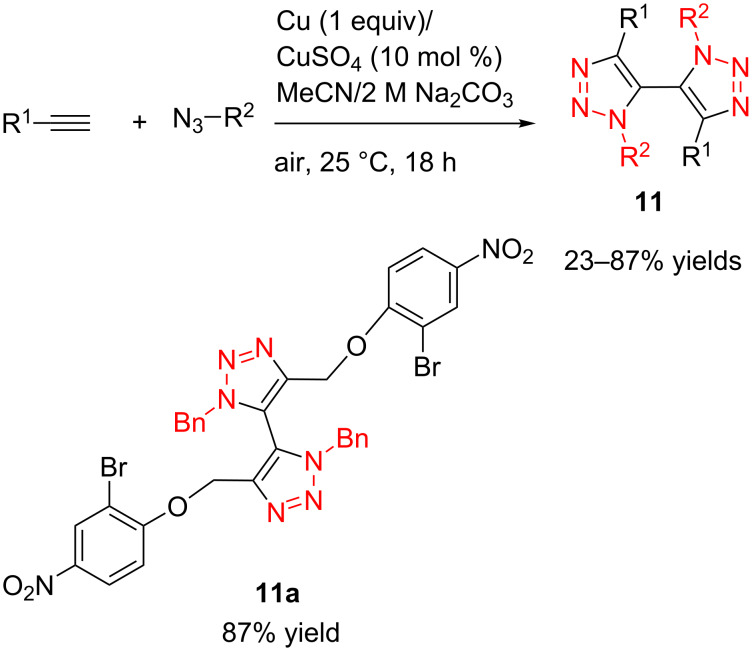
The synthesis of 5,5'-bitriazoles.

In 2007, Burgess and Angell successfully developed an oxidative coupling method for the preparation of 5,5’-bitriazole [21]. In this work, they were able to perform this reaction of azides and terminal alkynes with moderate to high yield by using a 1:1 mixture of MeCN/2 M aqueous Na_2_CO_3_ solution at 25 °C for 18 h in the presence of a catalytic amount of CuSO_4_ (10 mol %) and one equivalent of Cu powder (Scheme 5). Obviously, this method suffered from the drawback that a stoichiometric amount of Cu powder is required to achieve the highest activity.

**Scheme 6:**
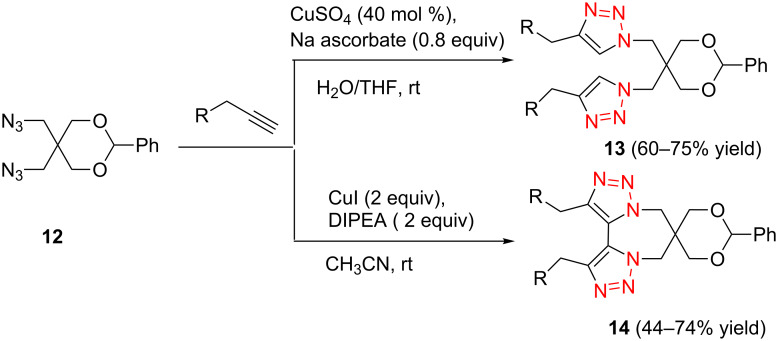
The synthesis of bistriazoles and cyclic 5,5^’^-bitriazoles under different catalytic systems.

In 2010, Nandurdikar et al. linked the two (or four) molecules of NO donor prodrugs together through the triazole spacers [22], which has potential application as NO-releasing materials. They first prepared the benzylidene-protected 2,2-di(azidomethyl)propane-1,3-diol containing the conformational strain. They also investigated the click reactions with various alkynes under different conditions (Scheme 6), and found that: (1) when catalyzed with CuSO_4_/Na ascorbate in THF/H_2_O, the reaction provided the normal bistriazoles **13** with moderate to good yield (60–75%). (2) By using CuI and DIPEA in acetonitrile to perform the reaction, the sequential, CuAAC oxidative coupling gave the cyclic 5,5’-bitriazoles **14** as the major product (44–74%). They demonstrated that both the reaction conditions and the conformational effect are beneficial for the formation of the 5,5'-bitriazoles.

**Scheme 7:**
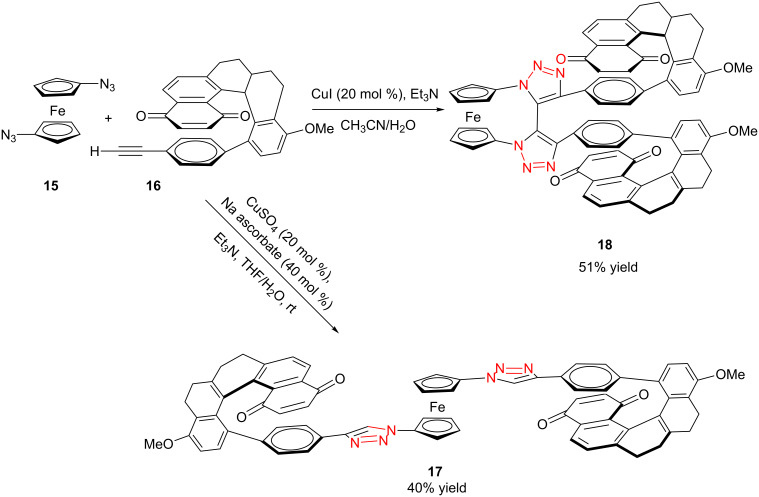
The double CuAAC reaction between helicenequinone and 1,1^’^-diazidoferrocene.

Similarly, Urbano and co-workers performed the CuAAC reaction of 1,1’-diazidoferrocene with ethynyl [5]-helicenequinone [23], and found the open chain bistriazolylphenyl-helicenequinone **17** could be obtained in good yield when CuSO_4_/sodium ascorbate was used in THF/H_2_O (Scheme 7). However, the cyclic 5,5'-bitriazole **18** was achieved as the major product when CuI/Et_3_N was used in CH_3_CN.

**Scheme 8:**
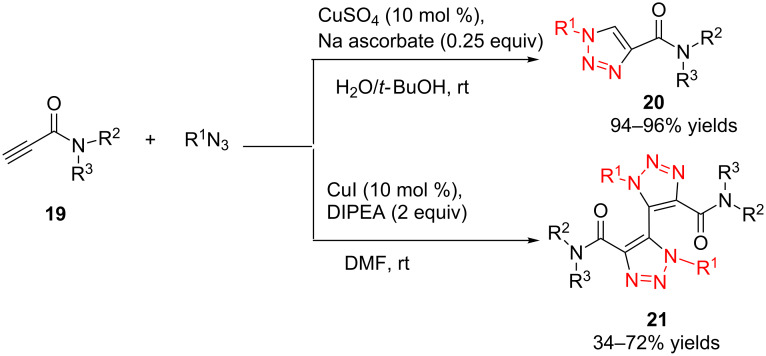
The synthesis of 1,2,3-triazoles and 5,5’-bitriazoles from acetylenic amide.

In 2011, Cuevas-Yañez and co-workers demonstrated that temperature was the key factor for the formation of 5,5’-bitriazole [24]. In that work, it was found that the desired bitriazoles could be obtained in low to moderate yield when the CuAAC reaction was performed at low temperature (−35 °C) and high NaOH concentration. Subsequently, Jeon and co-workers reported a synthetic method for the construction of 5,5’-bitriazoles by using CuI together with 2 equiv of DIPEA [25]. The authors further demonstrated that the use of a base was also an important factor for the appropriate bitriazole yield. However, Jeon’s method worked well only when the alkynes contained propargylic ethers and acetylenic amides (Scheme 8). Generally speaking, most of the above-mentioned oxidative coupling-click dimerizations merely provided the desired 5,5’-bitriazole in low yield when the alkynes or azides were linked directly with a hindered group or an aromatic moiety.

**Scheme 9:**
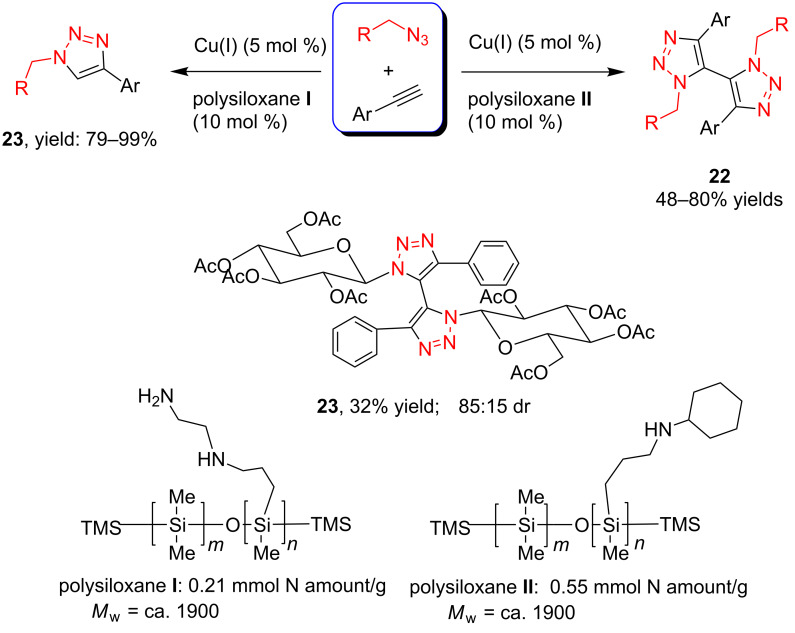
The amine-functionalized polysiloxane-mediated divergent synthesis of trizaoles and bitriazoles.

**Scheme 10:**
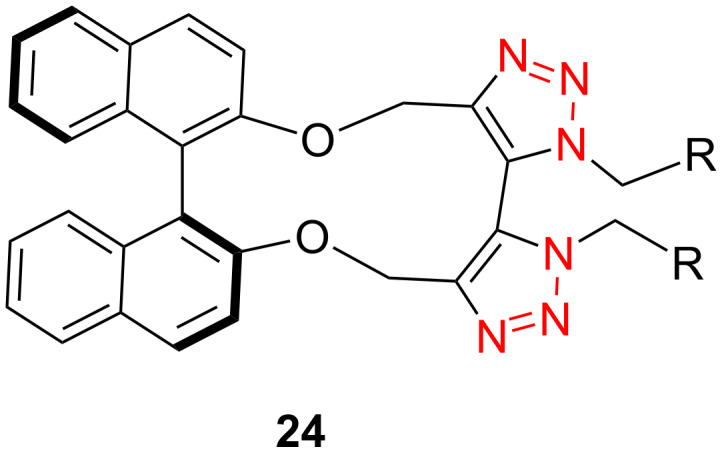
The cyclic BINOL-based 5,5’-bitriazoles.

In 2012, Xu and co-workers disclosed that both the secondary and primary amine-functionalized polysiloxanes were good ligands for the copper-catalyzed Huisgen reaction of organic azides and alkynes [26]. This applied especially when the reaction was catalyzed by CuCl and mediated by the secondary amine-functionalized polysiloxanes at 0 °C in CH_2_Cl_2_, where the symmetrically 5,5’-coupled bitriazole was obtained as the major product (Scheme 9). All the alkynes directly linked with the aromatic moiety provided the desired bitriazoles in moderate to good yield. We believe that the key copper monotriazole intermediate formed after the first CuAAC reaction was stabilized by the secondary amine-functionalized polysiloxane, which further led to the formation of the bitriazole **22**. The chiral D-glucopyranosyl azide and phenylacetylene reacted under CuI and the secondary amine-functionalized polysiloxane catalytic system, where the desired chiral bitriazole **23** was obtained with good diastereoselectivity (85:15). Subsequently, the authors used this method to construct the novel, cyclic 5,5’-bitriazole **24** from binaphthol [27] (Scheme 10). Notably, this type of compound showed high selectivity over the recognition of I^−^, possibly due to the formation of a charge-transfer complex between the I^−^ and the electron-deficient triazole ring.

### Bistriazoles formed through spacers

#### Bistriazole synthesis with dialkyne spacers

In the past years, bistriazoles with dialkyne spacers have gained significant attention for their potential application in supramolecular chemistry, pharmaceutical chemistry, biological chemistry and organometallic chemistry. The construction of bistriazoles from dialkynes is now well-developed, and three main methods are reported: (1) the one-pot CuAAC reactions of the terminal dialkynes with two equiv of the organic azides, providing the corresponding bistriazoles, which is the most popular method for the synthesis of bistriazoles with dialkyne spacers. (2) The second protocol is the sequential CuAAC–deprotection–CuAAC reaction for the construction of the bistriazoles. In general, the trialkylsilyl group was used as a temporary masking group for one of two alkyne moieties. Thus, this method provides the possibility of one molecule bearing two alkynes reacted with two different organic azides. (3) The third method involves the utilization of the substrate bearing two alkyne moieties with different reactivity in the successive Huisgen cycloaddition reactions: Huigsen reaction of the activated alkyne with the first azide and the CuAAC reaction of the nonactivated alkyne with another azide, leading to the corresponding bistriazoles.

**Scheme 11:**
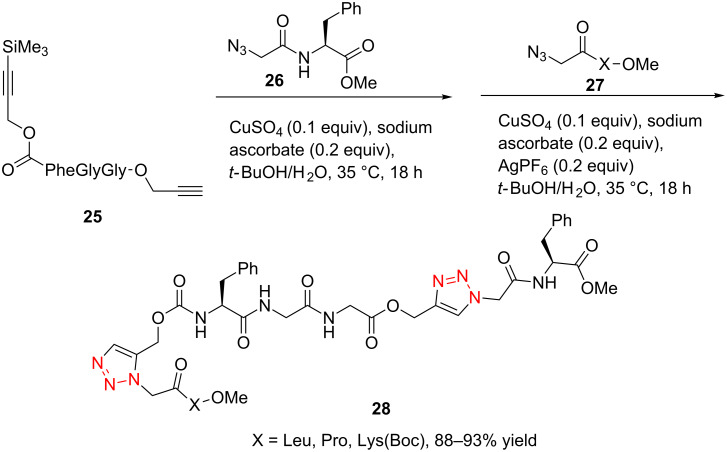
The one-pot click–click reactions for the synthesis of bistriazoles.

In 2006, Aucagne and Leigh reported the synthesis of the TMS-alkyne and terminal-alkyne bis-functionalized tripeptide [28]. Here they used the CuAAC reaction of the terminal-alkyne-containing tripeptide **25** with the azide-containing dipeptide **26** in *t*-BuOH/H_2_O, catalyzed by CuSO_4_/sodium ascorbate, providing the first triazole-bearing intermediate (Scheme 11). They then performed the Ag(I)-catalyzed deprotection of the TMS-protected alkyne moiety, followed by another CuAAC reaction of the unmasked terminal alkyne with the second azide, giving the desired bistriazole-linked pseudononapeptides **28** in good yield (88–93%) .

**Scheme 12:**
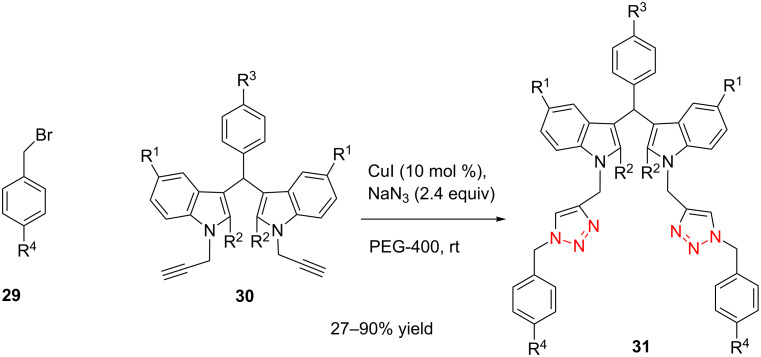
The synthesis of bis(indolyl)methane-derivatized 1,2,3-bistriazoles.

In 2009, Perumal and co-workers developed a one-pot methodology [29] for the multicomponent cycloaddition of sodium azide, benzyl bromides and various *N*-propargylated bis(indolyl)arylmethanes **30** catalyzed by CuI. It was proved that this multicomponent CuAAC reaction proceeded well in PEG-400 among various solvents, mainly due to the hydrophilic and hydrophobic character of the PEG-400. As shown in Scheme 12, the CuAAC gave the desired bistriazoles **31** in good to excellent yield when electron-withdrawing groups were present in the benzyl bromides. Notably, the authors determined that all the compounds obtained showed potential biological activity.

**Scheme 13:**
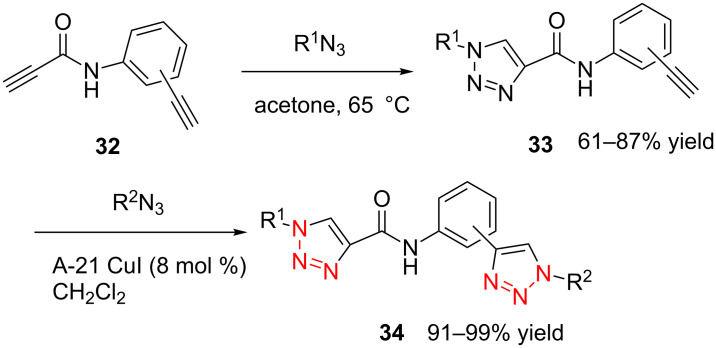
The sequential, chemoselective preparation of bistriazoles.

Later, in 2011, Girard et al. demonstrated that the dialkynes with functional groups could react smoothly with various organic azides without the protection–deprotection step [30]. Initially, they first chose *N*-propargylpropiolamide **32** as the substrate and found the alkyne group with neighboring electron-withdrawing amide carbonyl was reacting exclusively with the organic azide under catalyst-free reaction conditions (with or without a solvent at room temperature) to give the mono-triazole intermediate **33** in good yield. Then the nonactivated terminal alkyne reacts with another azide, catalyzed by an Amberlyst A-21/CuI system in CH_2_Cl_2_ to form the second triazole ring with high yield (Scheme 13). In this work, the authors further extended this method to the propiolamide, which was derived from *meta*- and *para*-ethynylaniline, where both of the substrates worked well and the desired bistriazoles **34** could be obtained by a simple trituration and filtration procedure in good yield.

**Scheme 14:**
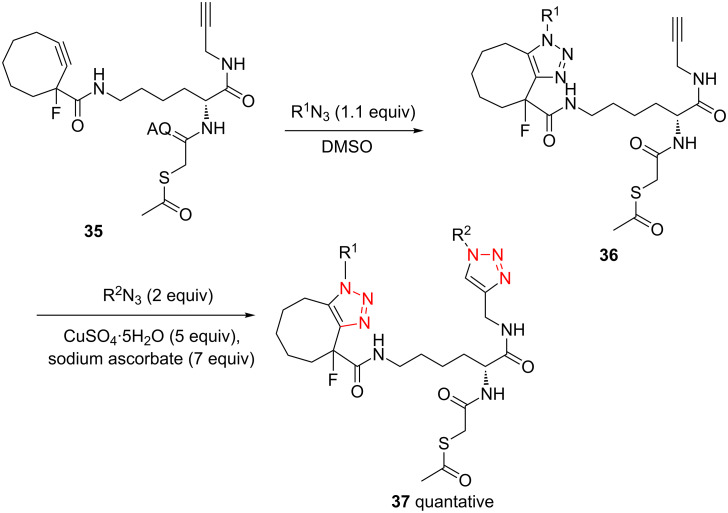
The sequential SPAAC and CuAAC reaction for the preparation of bistriazoles.

The strain-promoted azide-alkyne cycloaddition (SPAAC) reaction could be well-performed without a Cu(І) catalyst. Such reactions are ideal for bioconjugation where no additional metal is required [31]. In 2012, Beal and co-workers incorporated the terminal alkyne and the activated cyclooctyne with two amide formation reactions [32], and then the SPAAC reaction was performed between the cyclooctyne **35** and the azides under mild conditions without the Cu(І). Notably, the CuAAC reaction between the terminal alkyne of **36** and various azides provided the desired bistriazoles **37** in good yield (Scheme 14).

**Scheme 15:**
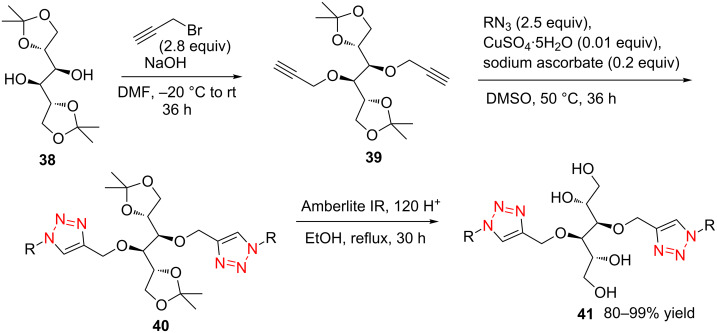
The synthesis of D-mannitol-based bistriazoles.

Bistriazoles have potential application in the synthesis of surfactants after introducing the carbohydrate moiety. In 2012, Mohammed and co-workers selected the commercially available D-mannitol **38** as the starting material [33] in this reaction. After protection and introduction of the two alkyne groups to give dialkyne **39**, the copper-catalyzed cycloaddition occurred smoothly with the different long chain alkyl azides under the optimized reaction conditions. This provided the desired bistriazoles **40** in good yield (Scheme 15). It should be noted that the deprotection of bistriazoles **40** gives the gemini surfactants **41** (with two hydrophobic arms and two hydrophilic heads) in high yield.

**Scheme 16:**
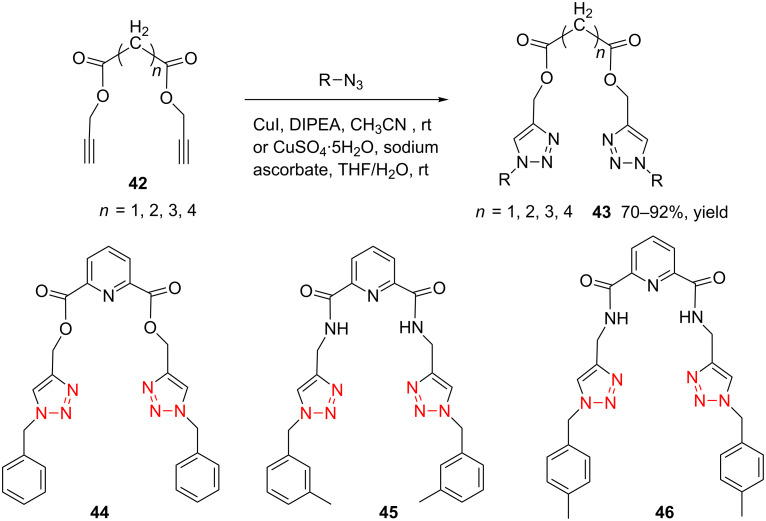
The synthesis of ester-linked and amide-linked bistriazoles.

In 2012, Kaushik and co-workers prepared a series of ester-linked dialkynes from the acid dichloride and propargyl alcohol in the presence of DMAP [34]. Here, the CuAAC reaction between the dialkynes **42** and the azides provided the desired bistriazoles **43** in good yield (70–92%, Scheme 16). Interestingly, the antimicrobial activity studies revealed that compound **44** showed the highest activity against *B. subtilis* and *E. coli* due to the presence of a rigid pyridine nucleus. The authors further prepared various amide-linked bistriazoles by a three-component one-pot reaction of the amide-linked dialkynes, benzyl bromides and sodium azide catalyzed by CuSO_4_·5H_2_O and sodium ascorbate in DMF [35–36]. All the obtained compounds were evaluated for in vitro cytotoxicity against a panel of five human cancer cell lines, where compounds **45** and **46** displayed the highest and broadest spectrum activity against all five cancer cell lines under study (Scheme 16).

**Scheme 17:**
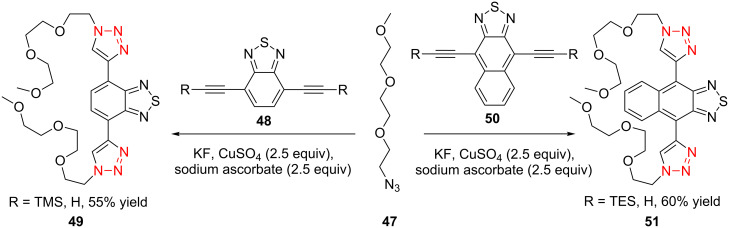
The synthesis of acenothiadiazole-based bistriazoles.

The formation of triazole functional groups can work as a powerful auxochrome. In 2010, Bunz and co-workers used the TMS-protected diethynylbenzothiadiazoles **48** and **50** as the source of dialkynes. The CuAAC reaction was carried out with ethylene-glycol-functionalized azide **47** in the presence of CuSO_4_ and sodium ascorbate, providing the benzo-thiadiazole-based bistriazole (**49** and **51**, Scheme 17) [37]. With the aid of the hydrophilic character of the ethylene glycol group, both of the bistriazoles can be endowed with water solubility, and can effectively bind Cu(II) and Ni(II) in water.

**Scheme 18:**
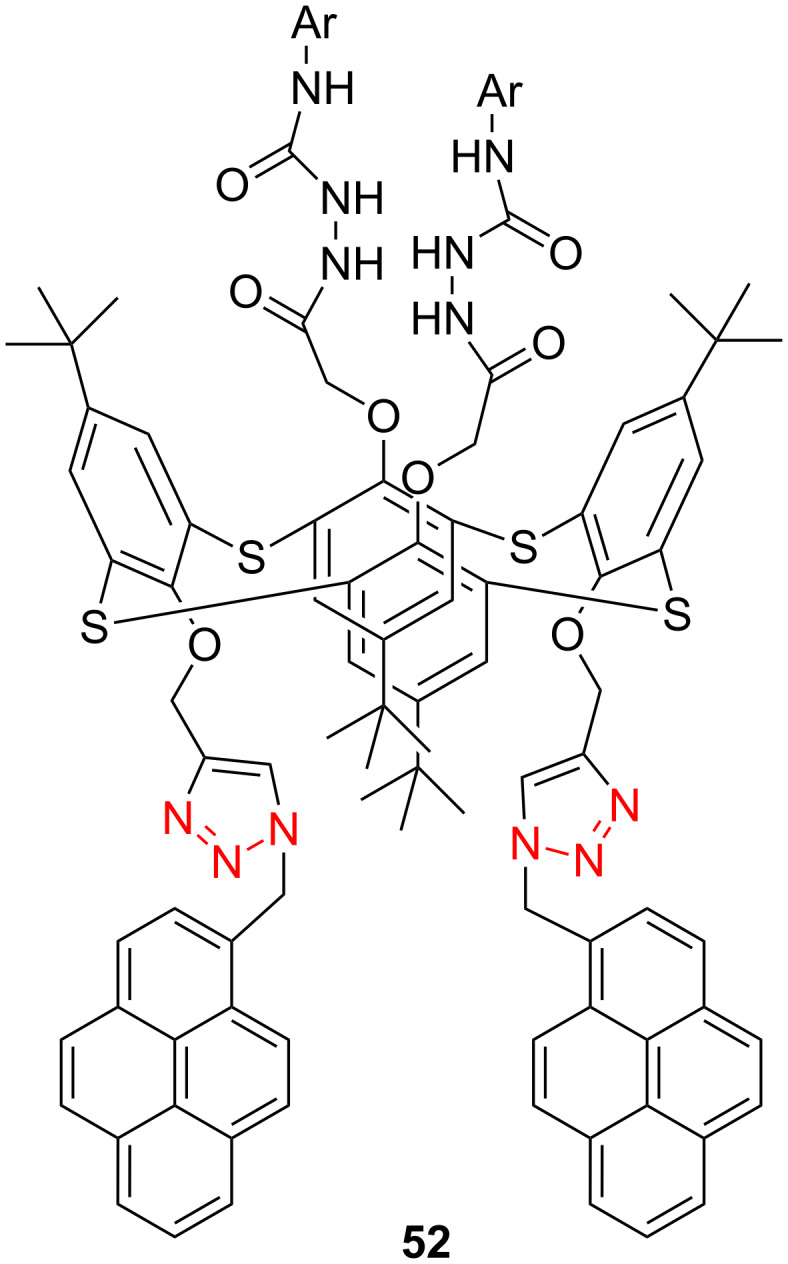
The pyrene-appended thiacalix[4]arene-based bistriazole.

The thiacalix[4]arenes are sulfur-bridged analogs of calix[4]arenes, which have potential application in the molecular recognition of cationic, anionic or neutral molecules. In this regard, Yamato et al. incorporated two urea moieties possessing various aryl groups and two pyrene-appended triazole rings at the opposite side of the thiacalix[4]arene cavity [38]. The authors found that receptor **52** (Scheme 18) could selectively bind Cl^−^ through hydrogen bonding interaction with the urea NH protons, and **52** can also bind with Ag^+^ through complexation with the pyrene-appended bistriazole.

**Scheme 19:**
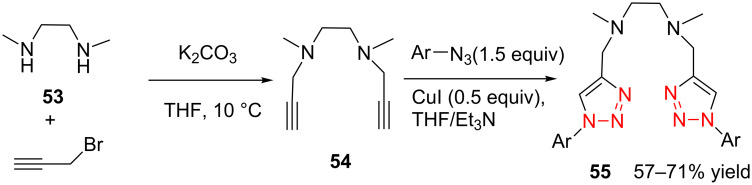
The synthesis of triazole-based tetradentate ligands.

In homogeneous catalysis, functional ligands often play a key role in transition metal catalysis. Accordingly, the bistriazole derivatives could provide promising alternatives to bipyridine ligands because of their powerful nitrogen-centered coordination. In this context, Hao and co-workers have reported bistriazole-based N4 tetradentate ligands that were prepared by two CuAAC reactions in a one-pot procedure [39]. As shown in Scheme 19, these ligands exhibited good coordination properties to various metals, and the corresponding Mn(II) complexes showed good catalytic activity for the epoxidation of various aliphatic terminal olefins.

**Scheme 20:**
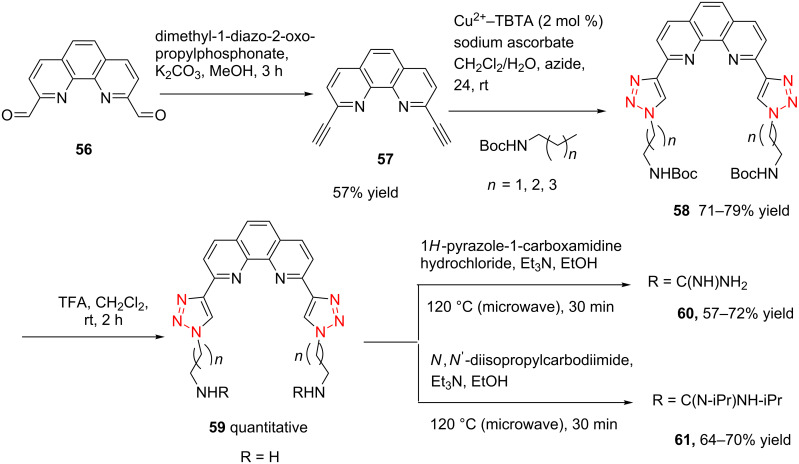
The synthesis of phenanthroline-2,9-bistriazoles.

Very recently, Ulven and co-workers reported the synthesis of triazole-linked phenanthroline ligands. They were obtained by the following steps: (1) 1,10-phenanthroline-2,9-dicarbaldehyde (**56**) was treated with the Ohira–Bestmann reagent to provide the corresponding dialkyne **57**; (2) Dialkyne **57** was reacted with different azides catalyzed by the Cu(II)–TBTA complex and sodium ascorbate in a bi-phasic system of CH_2_Cl_2_/H_2_O as the solvent, giving the desired bistriazoles **59** in good yield; (3) Deprotection of the N-Boc group with TFA, and the obtained primary amines was transformed to the corresponding primary guanidine or diisopropylguanidine analogs (**60** and **61**, Scheme 20) [40], which could be used as potential G4 DNA ligands with high selectivity over duplexed DNA.

Similar to the above strategies or methods, a number of researchers have developed various dialkyne substrates with varied spacers. As shown in Table 1, the reaction conditions are summarized for comparison, including the catalysts, the solvents, and the application of the bistriazoles.

**Table 1:** Previous reports on the copper-catalyzed Huisgen cycloaddition to bistriazoles with spacers.

Cu source	Solvent	Spacer	Azide	Application

CuSO_4_·5H_2_O, sodium ascorbate [41]	DMSO/H_2_O	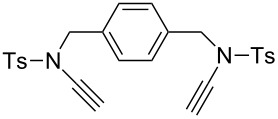	BnN_3_	–
bioClick conditions [42]	–	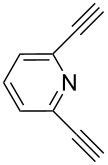	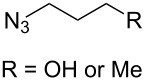	Complexation
CuSO_4_·5H_2_O, sodium ascorbate [43]	CH_2_Cl_2_/H_2_O	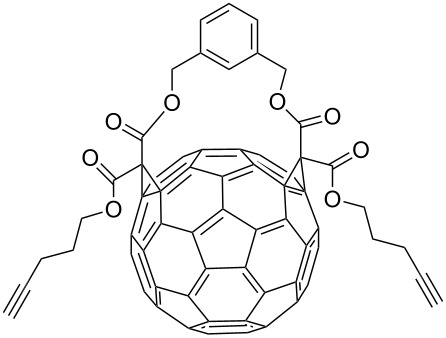	6 azidosugars	–
A-21-CuI [44]	CH_2_Cl_2_	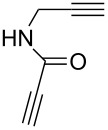	R–N_3_, R = Bn,CH_2_CO_2_Et, (CH_2_)_3_OAc, (CH_2_)_3_OH	–
Cp*RuCl(COD) [45]	THF	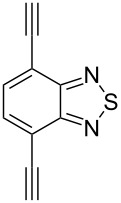	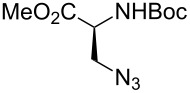	Chemical sensors
CuSO_4_, sodium ascorbate [46]	H_2_O/*t-*BuOH	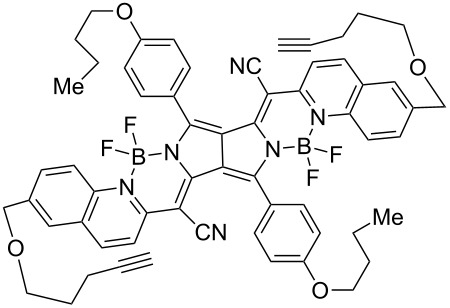	3 azides	Biological imaging
[Cu(CH_3_CN)_4_] (PF_6_) [47]	CH_2_Cl_2_	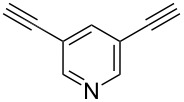	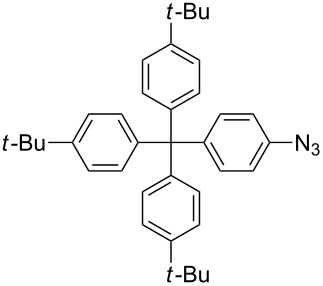	Anion recognition
CuI [48]	THF	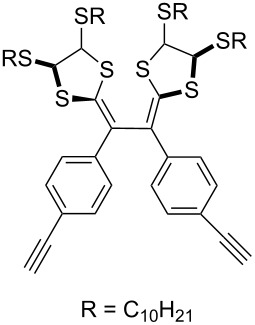	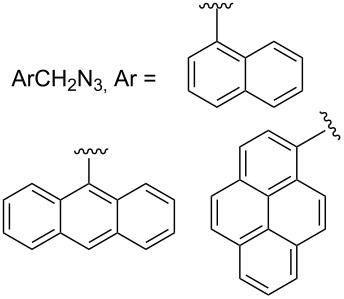	Receptors for fullerenes
CuSO_4_·5H_2_O, sodium ascorbate [49]	H_2_O/*t-*BuOH	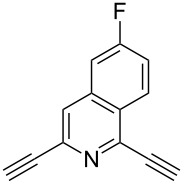	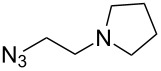	Chemical sensors
CuSO_4_·5H_2_O, sodium ascorbate [50]	H_2_O/*t-*BuOH	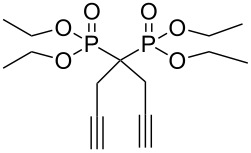	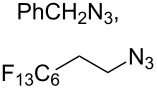	
CuSO_4_, sodium ascorbate [51]	H_2_O/*t-*BuOH	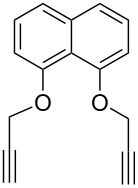	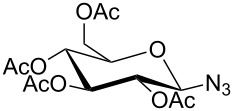	Chemical sensor
CuI [52]	–	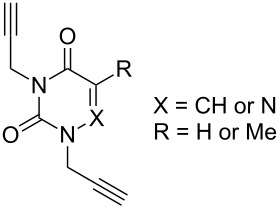	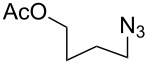	Biological activity
CuSO_4_·5H_2_O, sodium ascorbate [53]	CH_2_Cl_2_/H_2_O	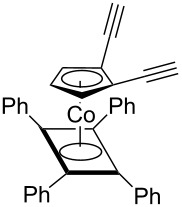	PhCH_2_N_3_	–
CuSO_4_·5H_2_O, sodium ascorbate [54]	DMF/H_2_O	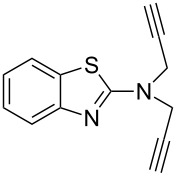	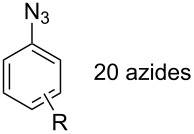	Hemolitic activity
CuSO_4_·5H_2_O, sodium ascorbate [55]	DMF/H_2_O	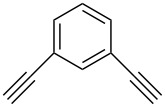	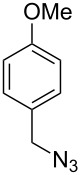	Complexation
CuSO_4_·5H_2_O, sodium ascorbate [56]	EtOH/H_2_O	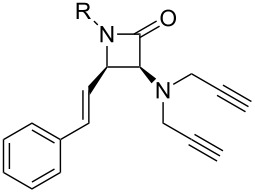	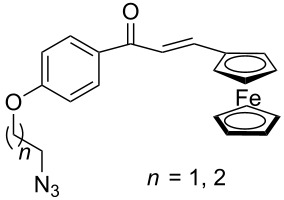	Antitubercular activity
CuSO_4_·5H_2_O/Cu [57]	CH_3_CN	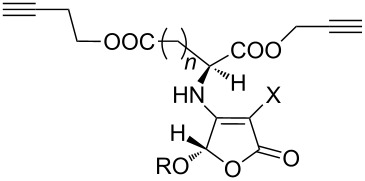	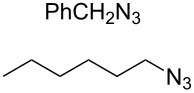	–
CuSO_4_, sodium ascorbate [58]	H_2_O/*t-*BuOH	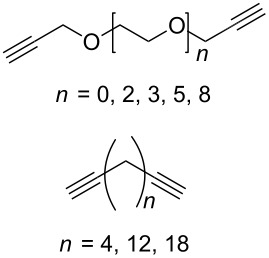	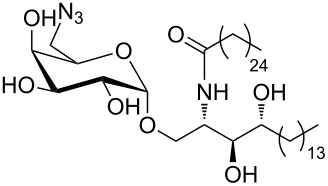	Biological activity
CuI [59]	THF/H_2_O	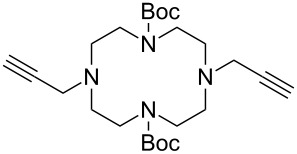	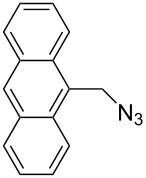	Chemical sensor
CuSO_4_·5H_2_O, sodium ascorbate [60]	H_2_O/*t-*BuOH	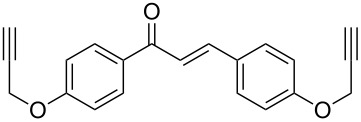	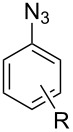	Antioxidant, antifungal activity
CuSO_4_, sodium ascorbate [61]	CH_2_Cl_2_/*t-*BuOH	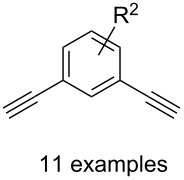	R^1^N_3_	Anion bonding
CuI [62]	CH_3_CN	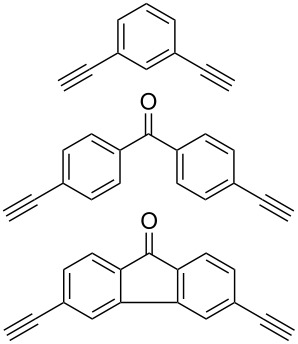	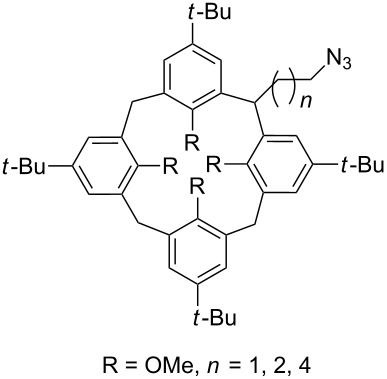	Chemical sensors
Cu(PPh_3_)_3_Br [63]	THF	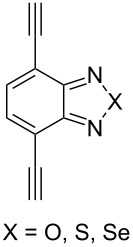	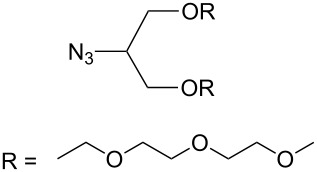	Chemical sensors

#### Bistriazole synthesis with diazide spacers

The spacer-linked bistriazoles could also be prepared by the CuAAC reaction of the in situ generated diazides with substitued alkynes. There are mainly two types of methods for the construction of bistriazoles from diazides: (1) Starting from the substrate-containing good-leaving groups, the diazides could be generated in situ by nucleophilic addition with NaN_3_, and then the double CuAAC reactions could give the desired bistriazoles. (2) Starting from a substrate containing one azide functionality and another suitable functional group, the CuAAC reaction was performed with a terminal alkyne to provide the monotriazole compound. Subsequently, the suitable functional group was transformed into the corresponding azide for the next CuAAC reaction to give the desired bistriazoles.

**Scheme 21:**
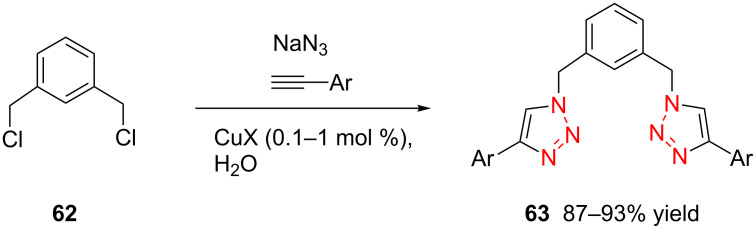
The three-component reaction for the synthesis of bistriazoles.

In 2007, Wang and co-workers demonstrated that the one-pot three-component reaction of *ortho*- and *meta*-bis(chloromethyl)benzene (**62**), sodium azide, and terminal alkynes, catalyzed by CuX in water could provide the corresponding 1,4-disubstitued bistriazoles **63** in excellent yield [64] (Scheme 21). They found that the bistriazole could be formed during the Huisgen reaction, in which the reaction was efficiently promoted by the catalytic amount of the Cu(I) salts. For example, the three-component reaction could reach completion in the presence of only 0.2 mol % of Cu(I).

**Scheme 22:**
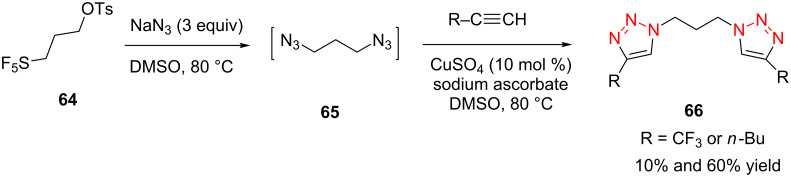
The one-pot synthesis of bistriazoles.

In 2010, Shreeve et al. reported that the diazides **65** could be generated in situ by the nucleophilic substitution of SF_5_ and OTs groups wtih 3 equiv of NaN_3_, followed by the CuAAC reaction with aliphatic alkynes, providing the desired bistriazoles **66** in moderate yield (Scheme 22) [65]. However, when they choose the substrate SF_5_-ethylbromide, only trace amounts of the triazole-containing compound was obtained even after 18 h at 60 °C due to the poor leaving ability of the Br^−^ compared to the OTs group.

**Scheme 23:**
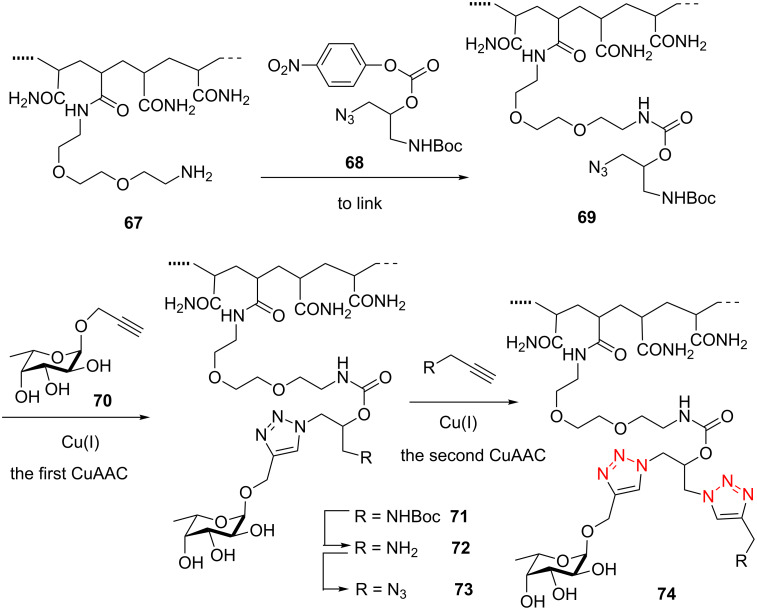
The synthesis of polymer-bearing 1,2,3-bistriazole.

In 2011, Bundle et al. reported the double-click method for the formation of disymmetric bistriazoles [66]. The authors introduced the azide group (**69**) by the coupling of the protected amine functionality to the polymer substrate for the first CuAAC reaction (Scheme 23). Then, the treatment of the amine-containing mono-triazole intermediate **71** with the diazo transfer agent (imidazole-1-sulfonyl azide) was performed to convert the amine group into the corresponding azide group, which provided a polymeric substrate for the second CuAAC reaction to give the desired bistriazoles (Scheme 23).

**Scheme 24:**
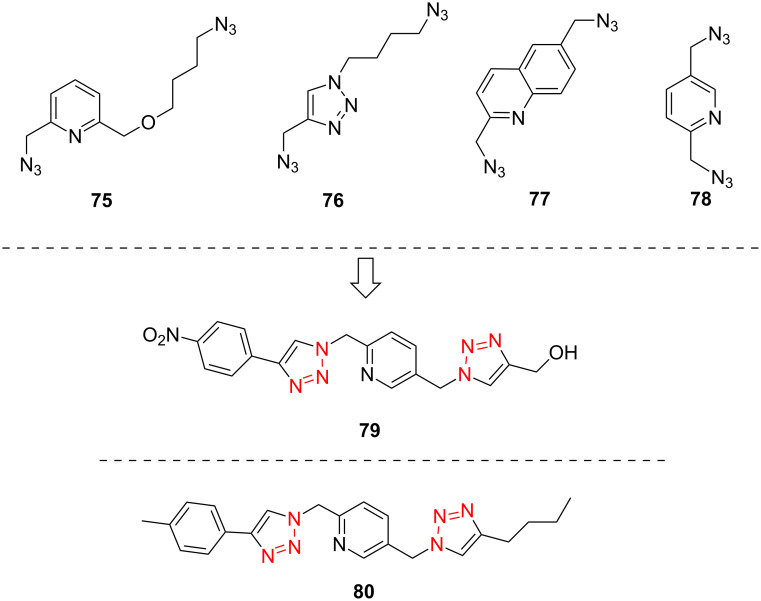
The synthesis of bistriazoles via a sequential one-pot reaction.

In 2009, Zhu and co-workers found that copper(II) acetate (Cu(OAc)_2_) could catalyze the Huisgen alkyne-azide cycloaddition reactions without the addition of the reducing agents and could be produced in high yield when the substrate contains the chelating azide group [67]. Then they synthesized the bifunctional compounds with chelating azide groups and nonchelating azide groups (compounds **75**–**78**, Scheme 24) [68], by adding the Cu(OAc)_2_. This promoted the Huisgen cycloaddition of the chelating azide with the terminal alkyne, providing the mono-triazole intermediates. Then, the introduction of the second alkyne, together with sodium ascorbate as the reducing agent, gave the desired bistriazoles in high yield. By mixing the two alkynes with different reactivities with the diazide, they developed a sequential one-pot method for the construction of the bistriazoles (**79**, **80**, etc.).

Notably, although there are many examples of the formation of spacer-linked bistriazoles from the diazide substrates, these bistriazole products were obtained with almost the same strategy as previously reported. These works are listed in Table 2 together with the reaction conditions and the applications of the corresponding bistriazoles.

**Table 2:** Previous reports on the copper-catalyzed Huisgen cycloaddition to bistriazoles with spacers.

Cu source	Solvent	Spacer	Alkyne	Application

Cu(OAc)_2_, sodium ascorbate [69]	H_2_O/*t-*BuOH	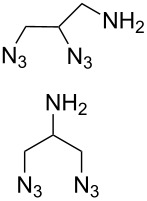	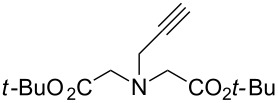	Complexation
CuI [70–71]	THF/H_2_O	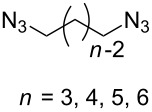	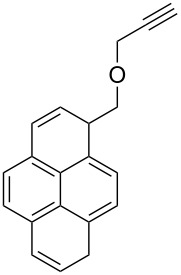	Chemical sensor
CuSO_4_·5H_2_O, sodium ascorbate [72]	DMF/H_2_O	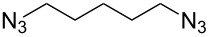	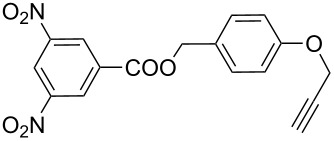	Supermolecular chemistry
CuSO_4_·5H_2_O, sodium ascorbate [73]	DMF	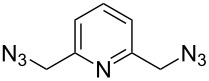	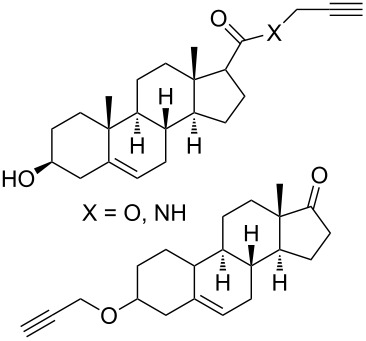	Cytotoxic activity
[74]	CHCl_3_	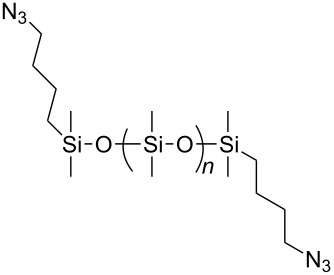	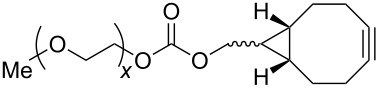	Polymer chemistry
CuSO_4_·5H_2_O, sodium ascorbate [75]	THF/H_2_O	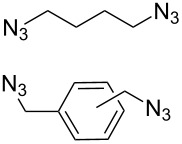	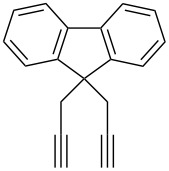	–
CuSO_4_·5H_2_O, sodium ascorbate [76]	H_2_O/*t-*BuOH	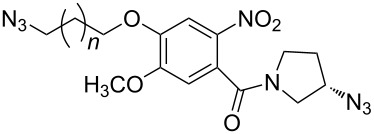	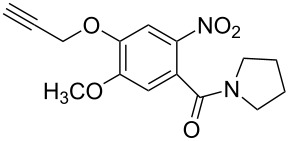	DNA binding
CuSO_4_·5H_2_O, sodium ascorbate [77]	THF/H_2_O	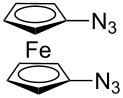	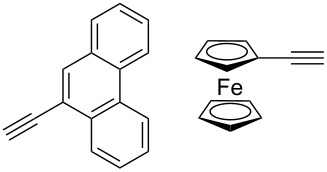	Receptor
CuSO_4_·5H_2_O, sodium ascorbate [78]	DMF/H_2_O	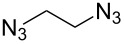	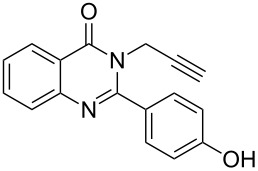	Molecular recognition
Cu(OAc)_2_/Cu [79]	DMSO	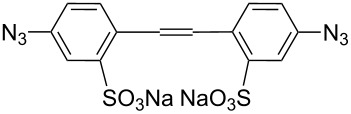	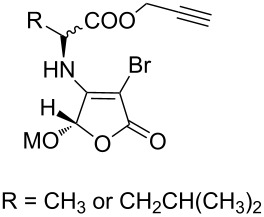	Fluorescence brightening agents
CuSO_4_/Cu [80]	EtOH	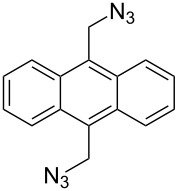	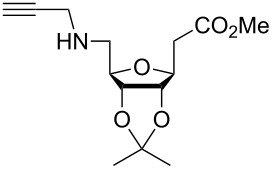	Chemical sensor
CuI [81]	CH_2_Cl_2_/MeOH	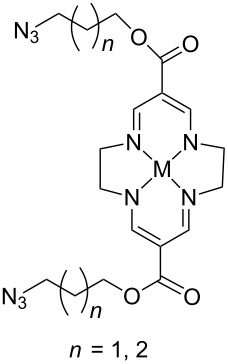	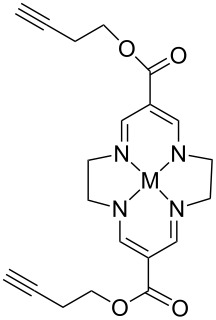	Electroactive receptor
Cu(OAc)_2_ sodium ascorbate [82]	*t-*BuOH	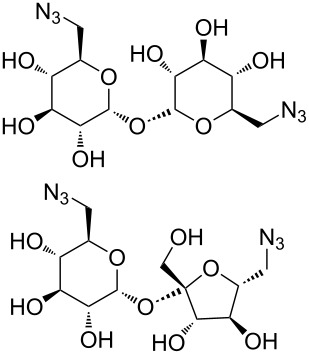	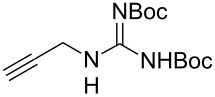	Biological activity

## Conclusion

During the past 15 years, the CuAAC reaction has become a powerful tool for the synthesis of a large number of 1,4-disubstituted 1,2,3-triazoles and has led to applications in almost every field of chemistry and biochemistry. In this review, we summarized the recent progress of the CuAAC reaction, together with various popular related reactions, which have unexpected potential to yield the 4,4'-, 5,5'-bitriazoles or spacer-linked bistriazoles. Nevertheless, as compared to the classic spacer-linked bistriazoles, the chemistry of 4,4'-, and 5,5'-bitriazoles (both their preparation and application) are still in their infancy. Although we restricted this review to describe only those having a bistriazole backbone, one can easily obtain a glimpse into the huge potential of bistriazoles in the broad sense when added to all other substitution possibilities. This topic, although already widely studied in the past years, is still continuously evolving and regularly brings new possibilities in click chemistry. We feel that this compilation will be beneficial to design practical approaches and better routes to improve the existing routes for the synthesis of synthetically useful bi- and bistriazoles, and we expect that the pace of discovery of the application of bi- and bistriazoles in many fields will continue to increase for some time.

## References

[1] Huisgen R (1989). Pure Appl Chem.

[2] Huisgen R, Szeimies G, Möbius L (1967). Chem Ber.

[3] Bastide J, Hamelin J, Texier F, Ven V Q (1973). Bull Soc Chim Fr.

[4] Bastide J, Hamelin J, Texier F, Ven V Q (1973). Bull Soc Chim Fr.

[5] Alvarez R, Velazquez S, San-Felix A, Aquaro S, De Clercq E, Perno C-F, Karlsson A, Balzarini J, Camarasa M J (1994). J Med Chem.

[6] Genin M J, Allwine D A, Anderson D J, Barbachyn M R, Emmert D E, Garmon S A, Graber D R, Grega K C, Hester J B, Hutchinson D K (2000). J Med Chem.

[7] Brockunier L L, Parmee E R, Ok H O, Candelore M R, Cascieri M A, Colwell L F, Deng L, Feeney W P, Forrest M J, Hom G J (2000). Bioorg Med Chem Lett.

[8] Fan W-Q, Katritzky A R, Katritzky A R, Rees C W, Scriven E F V (1996). Comprehensive heterocyclic chemistry II.

[9] Tornøe C W, Christensen C, Meldal M (2002). J Org Chem.

[10] Rostovtsev V V, Green L G, Fokin V V, Sharpless K B (2002). Angew Chem, Int Ed.

[11] Moses J E, Moorhouse A D (2007). Chem Soc Rev.

[12] Finn M G, Fokin V V (2010). Chem Soc Rev.

[13] Ganesh V, Sudhir V S, Kundu T, Chandrasekaran S (2011). Chem – Asian J.

[14] Thirumurugan P, Matosiuk D, Jozwiak K (2013). Chem Rev.

[15] Tang W, Becker M L (2014). Chem Soc Rev.

[16] Meldal M, Tornøe C W (2008). Chem Rev.

[17] Monkowius U, Ritter S, König B, Zabel M, Yersin H (2007). Eur J Inorg Chem.

[18] Fiandanese V, Bottalico D, Marchese G, Punzi A, Capuzzolo F (2009). Tetrahedron.

[19] Doak B C, Scanlon M J, Simpson J S (2011). Org Lett.

[20] Aizpurua J M, Azcune I, Fratila R M, Balentova E, Sagartzazu-Aizpurua M, Miranda J I (2010). Org Lett.

[21] Angell Y, Burgess K (2007). Angew Chem, Int Ed.

[22] Oladeinde O A, Hong S Y, Holland R J, Maciag A E, Keefer L K, Saavedra J E, Nandurdikar R S (2010). Org Lett.

[23] del Hoyo A M, Latorre A, Diaz R, Urbano A, Carreño M C (2015). Adv Synth Catal.

[24] González J, Pérez V M, Jiménez D O, Lopez-Valdez G, Corana D, Cuevas-Yañez E (2011). Tetrahedron Lett.

[25] Kwon M, Jang Y, Yoon S, Yang D, Jeon H B (2012). Tetrahedron Lett.

[26] Zheng Z-J, Ye F, Zheng L-S, Yang K-F, Lai G-Q, Xu L-W (2012). Chem – Eur J.

[27] Wang C-Y, Zou J-F, Zheng Z-J, Huang W-S, Li L, Xu L-W (2014). RSC Adv.

[28] Aucagne V, Leigh D A (2006). Org Lett.

[29] Damodiran M, Muralidharan D, Perumal P T (2009). Bioorg Med Chem Lett.

[30] Elamari H, Meganem F, Herscovici J, Girard C (2011). Tetrahedron Lett.

[31] Kele P, Mezö G, Achatz D, Wolfbeis O S (2009). Angew Chem, Int Ed.

[32] Beal D M, Albrow V E, Burslem G, Hitchen L, Fernandes C, Lapthorn C, Roberts L R, Selby M D, Jones L H (2012). Org Biomol Chem.

[33] Mohammed A I, Abboud Z H, Alghanimi A H O (2012). Tetrahedron Lett.

[34] Lal K, Kumar A, Pavan M S, Kaushik C P (2012). Bioorg Med Chem Lett.

[35] Lal K, Kaushik C P, Kumar K, Kumar A, Qazi A K, Hamid A, Jaglan S (2014). Med Chem Res.

[36] Lal K, Kaushik C P, Kumar A (2015). Med Chem Res.

[37] Brombosz S M, Appleton A L, Zappas A J, Bunz U H F (2010). Chem Commun.

[38] Tomiyasu H, Shigyo N, Ni X-L, Zeng X, Redshaw C, Yamato T (2014). Tetrahedron.

[39] Hao E, Wang Z, Jiao L, Wang S (2010). Dalton Trans.

[40] Nielsen M C, Larsen A F, Abdikadir F H, Ulven T (2014). Eur J Med Chem.

[41] Zhang X, Li H, You L, Tang Y, Hsung R P (2006). Adv Synth Catal.

[42] Li Y, Huffman J C, Flood A H (2007). Chem Commun.

[43] Pereira G R, Santos L J, Luduvico I, Alves R B, Pereira de Freitas R (2010). Tetrahedron Lett.

[44] Elamari H, Jlalia I, Louet C, Herscovici J, Meganem F, Girard C (2010). Tetrahedron: Asymmetry.

[45] Ruan Y-B, Yu Y, Li C, Bogliotti N, Tang J, Xie J (2013). Tetrahedron.

[46] Zhou M, Zhang X, Bai M, Shen D, Xu B, Kao J, Ge X, Achilefu S (2013). RSC Adv.

[47] White N G, Beer P D (2013). Org Biomol Chem.

[48] Mulla K, Shaik H, Thompson D W, Zhao Y (2013). Org Lett.

[49] Midya G C, Paladhi S, Bhowmik S, Saha S, Dash J (2013). Org Biomol Chem.

[50] Skarpos H, Osipov S N, Vorob'eva D V, Odinets I L, Lork E, Röschenthaler G-V (2007). Org Biomol Chem.

[51] Hemamalini A, Mohan Das T (2013). New J Chem.

[52] Krim J, Taourirte M, Engels J W (2012). Molecules.

[53] Singh N, Metla B P R, Elias A J (2012). J Organomet Chem.

[54] Singh M K, Tilak R, Nath G, Awasthi S K, Agarwal A (2013). Eur J Med Chem.

[55] Scott S Ø, Gavey E L, Lind S J, Gordon K C, Crowley J D (2011). Dalton Trans.

[56] Kumar K, Carrère-Kremer S, Kremer L, Guérardel Y, Biot C, Kumar V (2013). Dalton Trans.

[57] Huo J-P, Lü M, Wang Z, Li Y (2012). Chin J Chem.

[58] Jervis P J, Moulis M, Jukes J-P, Ghadbane H, Cox L R, Cerundolo V, Besra G S (2012). Carbohydr Res.

[59] Xu H-R, Li K, Liu Q, Wu T-M, Wang M-Q, Hou J-T, Huang Z, Xie Y-M, Yu X-Q (2013). Analyst.

[60] Anthony P, Bashir N, Parveen R (2014). Asian J Pharm Sci.

[61] Asmus S, Beckendorf S, Zurro M, Mück-Lichtenfeld C, Fröhlich R, Mancheño O G (2014). Chem – Asian J.

[62] Fischer C, Weber E (2014). J Inclusion Phenom Macrocyclic Chem.

[63] Bryant J J, Lindner B D, Bunz U H F (2013). J Org Chem.

[64] Wang Z-X, Zhao Z-G (2007). J Heterocycl Chem.

[65] Huang Y, Gard G L, Shreeve J M (2010). Tetrahedron Lett.

[66] Guiard J, Fiege B, Kitov P I, Peters T, Bundle D R (2011). Chem – Eur J.

[67] Brotherton W S, Michaels H A, Simmons J T, Clark R J, Dalal N S, Zhu L (2009). Org Lett.

[68] Yuan Z, Kuang G-C, Clark R J, Zhu L (2012). Org Lett.

[69] Camp C, Dorbes S, Picard C, Benoist E (2008). Tetrahedron Lett.

[70] Hung H-C, Cheng C-W, Ho I-T, Chung W-S (2009). Tetrahedron Lett.

[71] Hung H-C, Cheng C-W, Wang Y-Y, Chen Y-J, Chung W-S (2009). Eur J Org Chem.

[72] Wei P, Yan X, Li J, Ma Y, Yao Y, Huang F (2012). Tetrahedron.

[73] Jurášek M, Džubák P, Sedlák D, Dvořáková H, Hajdúch M, Bartůněk P, Drašar P (2013). Steroids.

[74] Isaacman M J, Corigliano E M, Theogarajan L S (2013). Biomacromolecules.

[75] Rajesh R, Periyasami G, Raghunathan R (2010). Tetrahedron Lett.

[76] Kamal A, Shankaraiah N, Reddy C R, Prabhakar S, Markandeya N, Srivastava H K, Sastry G N (2010). Tetrahedron.

[77] Romero T, Orenes R A, Tárraga A, Molina P (2013). Organometallics.

[78] Karuturi R, Al-Horani R A, Mehta S C, Gailani D, Desai U R (2013). J Med Chem.

[79] Huo J-P, Luo J-C, Wu W, Xiong J-F, Mo G-Z, Wang Z-Y (2013). Ind Eng Chem Res.

[80] Huang H-J, Fang H-Y, Chir J-L, Wu A-T (2011). Luminescence.

[81] Mames I, Wawrzyniak U E, Woźny M, Bilewicz R, Korybut-Daszkiewicz B (2013). Dalton Trans.

[82] Westermann B, Dörner S, Brauch S, Schaks A, Heinke R, Stark S, van Delft F L, van Berkel S S (2013). Carbohydr Res.

